# The complete chloroplast genome sequence of Camellias (*Camellia fangchengensis*)

**DOI:** 10.1080/23802359.2017.1419086

**Published:** 2017-12-21

**Authors:** Yuan Liu, Yan Han

**Affiliations:** a Plant Germplasm and Genomics Center, Germplasm Bank of Wild Species in Southwest China, Kunming Institute of Botany, The Chinese Academy of Sciences, Kunming, China;; b Faculty of Life Science and Technology, Kunming University of Science and Technology, Kunming, China

**Keywords:** *Camellia fangchengensis*, chloroplast genome, Endangered species

## Abstract

*Camellia fangchengensis* is endemic to Fangcheng, Guangxi Province, China, and its populations have been shrinking. In the present study, we report the complete chloroplast genome of *C. fangchengensis* using HiSeq 2500 sequencing technology. The complete chloroplast genome length is 157,095 bp. The chloroplast genome has 43 tRNA genes, six rRNA genes, and 97 protein-coding genes. Moreover, 18 genes have multiple copies in the chloroplast genome. A total of 146 genes are present in the chloroplast genome. Maximum likelihood phylogenetic tree based on 11 complete chloroplast genomes revealed that *C. fangchengensis* is closely related to *C. pitardii*. The complete chloroplast genome of *C. fangchengensis* would help to conserving the precious natural populations.

*Camellia fangchengensis* is found in Fangcheng, Guangxi Province, China. The occurrence of this species is estimated to be less than 100 km^2^, consisting of only one location. The last known herbarium specimen was collected in 1994, and several botanists have failed to relocate the species in the wild at the type locality. The red list category is Critically Endangered B1ab(iii) (http://www.iucnredlist.org/details/62057104/0). The genus *Camellia* possesses significant economic values (Yang et al. 2013; Huang et al. 2014), and it provides excellent materials for studying the interspecific hybridisation in plant sciences (Yang et al. 2013). *Camellia fangchengensis* might become a good material for studying functional genomics and provide a valuable breeding resource in the genus of *Camellia*. The chloroplast genome information which has been applied in plant biology, phylogenetic inference and species identification (Kane et al. 2012; Ruhfel et al. 2014). However, a total of 14 complete chloroplast genomes in the genus *Camellia*, excluding that of *C. fangchengensis* have been reported (Shi et al. 2013; Yang et al. 2013; Huang et al. 2014).

In the present study, we assembled the complete chloroplast genome sequence of *C. fangchengensis* using Illumina HiSeq2500 sequencing technology. We have deposited the complete chloroplast genome sequence into GenBank database (Accession Number: MG198672).

Young leaves of *C. fangchengensis* were collected from International Camellia Species Garden (29°07′21.60″ N, 119°35′34.98″ E). The specimens have been preserved in the laboratory of the Kunming Institute of Botany, The Chinese Academy of Sciences, and the accession number is CF201709. Total genomic DNA was extracted from young leaves (10 g) by CTAB method (Doyle and Doyle 1987).

Pair-end sequencing (Library insert size: 500 bp) was performed using the HiSeq 2500 platform following the manufacturer’s protocol (Illumina, San Diego, CA, USA). Trimmomatic v0.22 (Bolger et al. 2014) was used to quality control. Totally, 59.72 M plastid reads were filtered from sequencing reads with a sufficient coverage, plastid reads were assembled to preliminary chloroplast genome by SOAPdenovo2 (Luo et al. 2012). Twenty-nine gaps were filled by the polymerase chain reaction (PCR)-based methods and GapFiller (Nadalin et al. 2012). The chloroplast genome was annotated by the software DOGMA (Wyman et al. 2004). A physical map of the chloroplast genome was prepared using the OGDRAW web server (Lohse et al. 2013).

We performed a phylogenetic analysis using nine complete chloroplast genomes of *Camellia* species and *Coffea arabica* as an out-group. Eleven complete chloroplast genomes were aligned by clustalw-mpi version 0.13 (Li 2003). Maximum likelihood analysis was implemented with MEGA v7.0.26 (Kumar et al. 2016).

The chloroplast genome of *C. fangchengensis* was 157,095 bp, with a circular structure. The length of inverted repeats (IR), large single copy (LSC), and small single copy (SSC) was 13,420, 130,675, and 19,354 bp, respectively.

The chloroplast genome of *C. fangchengensis* contained six rRNA genes, 43 tRNA genes, and 97 protein-coding genes. Among them, 11 protein-coding genes (*atpF*, *ndhA*, *ndhB*, *orf42*, *rpl2*, *rps12*, *rps7*, *ycf1*, *ycf15*, *ycf2*, and *ycf68*), five tRNA (*trnL-UAA*, *trnV-UAC*, *trnI-GAU*, *trnA-UGC*, and *trnI-GAU*), and three rRNA genes (*16SrRNA*, *23SrRNA*, and *5SrRNA*) had multiple copies. Annotation revealed that six tRNA genes, seven protein-coding genes and three rRNA genes were duplicated in the IR region. Totally, 146 genes were present in the chloroplast genome of *C. fangchengensis*, and the G + C content was 37.19% (A = 31.17%, T = 31.64%, G = 18.21%, and C = 18.98%).

The maximum likelihood phylogenetic tree showed that *C. fangchengensis* was closely related to *C. pitardii*. Five species of *Camellia* formed a single cluster (*C. grandibracteata*, *C. sinensis*, *C. leptophylla*, *C. petelotii*, and *C. impressinervis*). Four species of *Camellia* formed another cluster (*C. crapnelliana*, *C. azalea*, *C. pitardii*, and *C. fangchengensi*s). *Camellia taliensis* was located outside the clusters of *Camellia* (Figure 1).

**Figure 1.**
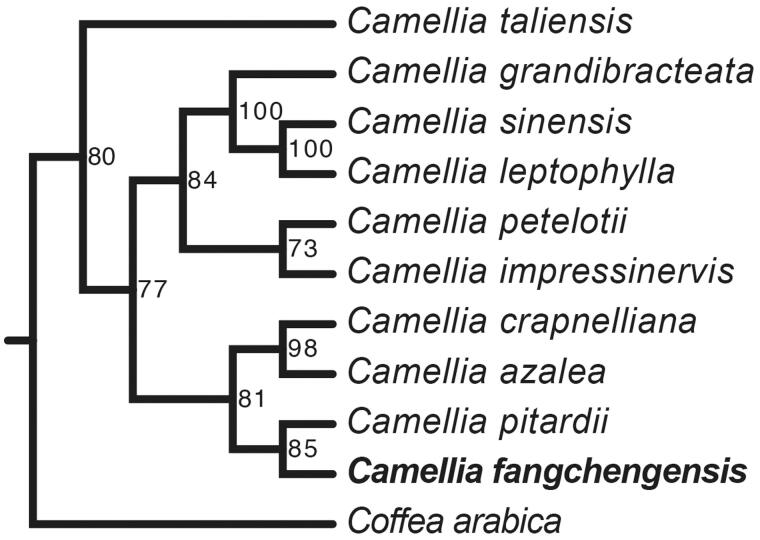
Maximum likelihood phylogenetic tree of *Camellia fangchengensis* based on 11 complete chloroplast genome sequences using *Coffea arabica* as an out-group. Numbers in the nodes are bootstrap values based on 1000 replicates. Accession numbers are listed as below: *Camellia azalea* NC_035574*, Camellia crapnelliana* NC_024541*, Camellia grandibracteata* NC_024659*, Camellia impressinervis* NC_022461*, Camellia leptophylla* NC_024660*, Camellia petelotii* NC_024661*, Camellia pitardii* NC_022462*, Camellia sinensis* NC_020019*, Camellia taliensis* NC_022264*, Coffea arabica* NC_008535.

The complete chloroplast genome of *C. fangchengensis* would provide information on phylogeny and species identity that would enhance the conservation strategies of *Camellia* in the future.
